# Clinical picture of diabetic ketoasidosis in Saiful Anwar Hospital Malang, Indonesia, 2005-2009

**DOI:** 10.1186/1687-9856-2013-S1-P28

**Published:** 2013-10-03

**Authors:** Harjoedi Adji Tjahjono

**Affiliations:** 1 Saiful Anwar Hospital Malang, Indonesia, 2005-2009

## Objective

To present the clinical appearance of diabetic ketoacidosis in patients were diagnosed in the Pediatric Department of Saiful Anwar Hospital between 2005 and 2009.

## Methods

A descriptive study of clinical appearance of diabetic ketoacidosis of 19 patients, 1-14 years of age, diagnosed in Pediatric Department of Saiful Anwar Hospital between 2005 and 2009.

## Results

From the 19 patients 58% were girls. 53% were between 5 and 10 years of age. 37% had an equal frequency between 400-600 mg/dl and 600-800 mg/dl of blood glucoses levels. 63% were undernourished. About 53% had moderate DKA. 53% patients had blood bicarbonate concentrations of 5-9,9 mmol/L. Blood osmolarity levels of >295-400 mOsm/l in 84% patients. 53% had blood potassium levels of 3,5-5 mmol/L. 42% patients had leukocyte counts of 10.000-<20.000/ul. The most common of symptoms of those patients were vomiting, abdominal pain, decreased of consciousness, seizures, diarrhea, decreased of body weight, polyuria, polydipsia, polyphagi and febris. The majority complained of polyuria about 16%. The majority patients of DKA had blood ketone levels of more than 5 mmol/l were 67%. 61% had ketonuri of 4+. The majority of those patients had anion gap levels of about 12.0-24.0 (63%).

## Conclusions

Of these 19 patients the majority were girls, aged between 5 and 10 years. The majority of patients had high blood glucose levels (between 400-600 mg/dl and 600-800 mg/dl). Most patients were undernourished and had moderate DKA, the majority had blood bicarbonate concentrations of about 5-9.9 mmol/l and high blood osmolarity levels (>295-400 mOsm/l). And most complained of polyuria and had high blood ketone levels (>5), potassium levels 3.5-5 mmol/l and leukocyte counts of 10.000-20.000 u/l.

